# Five weeks of dynamic finger flexor strength training on bouldering performance and climbing-specific strength tests. A randomized controlled trial

**DOI:** 10.3389/fphys.2024.1461820

**Published:** 2024-10-10

**Authors:** Atle Hole Saeterbakken, Erik Bratland, Vidar Andersen, Nicolay Stien

**Affiliations:** Department of Sport Food and Natural Sciences, Faculty of Education, Arts, and Sports, Western Norway University of Applied Sciences, Sogndal, Norway

**Keywords:** performance, finger flexor strength, rate of force development, resistance training, climbing, bouldering

## Abstract

The aim of the study was to examine the effects of a 5-week dynamic finger flexor strength training program on bouldering performance and climbing-specific strength tests. Advanced to elite level boulderers (n = 31) were randomized to a dynamic finger strength training group (DFS) or a control group (CON). The DFS training program consisted of 3 weekly sessions (3–5 sets, 4–10 repetitions per session). Both groups continued bouldering training as usual throughout the intervention period. Pre- and post-intervention measures included bouldering performance, maximal dynamic finger strength, isometric finger strength (peak and average force), and rate of force development (RFD). The DFS demonstrated greater improvement in dynamic finger strength (11.5%, 3.9 kg) than the CON (5.3%, 1.7 kg; *p* = 0.075, ES = 0.90), but there were no differences between the groups in 1RM (*p* = 0.075, ES = 0.67), bouldering performance (*p* = 0.39, ES = 0.35), isometric finger strength (*p* = 0.42–0.56, ES = 0.20–0.22) or RFD (*p* = 0.30, ES = 0.46). The DFS improved dynamic (*p* < 0.01, ES = 1.83) and isometric peak and average (*p* < 0.01, ES = 0.98, and *p* < 0.01, ES = 0.75, respectively) finger strength, while the CON only increased dynamic finger strength (*p* < 0.05, ES = 0.58). None of groups improved bouldering performance or RFD (*p* = 0.07–0.58). In conclusion, 5 weeks of DFS training improving dynamic strength to a greater extent than bouldering alone in addition to improving isometric finger strength among advanced boulderers. Isolated bouldering improved dynamic finger flexor strength, but importantly, increased finger strength (dynamic or isometric) did not improve bouldering performance.

## Introduction

Over 25 million participate in sport climbing worldwide (Saul et al., 2019) and the sport entered the Olympic program in Tokyo in 2021. Sport climbing includes multiple sub-disciplines, with lead climbing and bouldering being the most practiced and researched disciplines (Mundry et al., 2021; Stien et al., 2023). Although researchers have gained interest in climbing performance (Mundry et al., 2021; Stien et al., 2023; Langer et al., 2023; American College of Sports Medicine, 2009; Saeterbakken et al., 2024; Draper et al., 2021), optimizing performance and effects of various training methods are still primarily based on anecdotal and not scientific evidence.

Independent of climbing discipline, each climbing route has its own unique style and difficulty using different types and sizes of holds, in addition to a variety in the steepness of the wall and length of individual moves. Several studies have investigated determinant factors in climbing performance, identifying strength, strength endurance and rate of force development (RFD) of the finger- and shoulder girdle muscles as key factors discriminating performance levels in addition to flexibility, technical, and mental skills (Saul et al., 2019; Draper et al., 2021; Ginszt et al., 2023; Laffaye et al., 2016; MacLeod et al., 2007; Magiera et al., 2013; Mermier et al., 2000; Vereide et al., 2022; Balas et al., 2012). Importantly, specialized boulderers have demonstrated greater climbing-specific isometric and dynamic strength, RFD, and power than specialized lead climbers, while no differences are observed between disciplines in strength-endurance outcomes (Fanchini et al., 2013; Fryer et al., 2017; Laffaye et al., 2014; Stien et al., 2019).

Two systematic reviews and meta-analyses including 12 and 11 original intervention studies have examined the effects of climbing-specific resistance training (Stien et al., 2023; Langer et al., 2023). Together with three more recently published original studies (Devise et al., 2022; Vigouroux and Devise, 2024; Stien et al., 2024). These articles represent the climbing intervention that have been conducted. Notably, Langer et al. (Langer et al., 2023) categorized the training as climbing specific if the training consisted of lead climbing or bouldering, semi-specific when using methods such as fingerboard- or campus board training, and unspecific if the training consisted of traditional resistance training. Although training effects on climbing-specific tests have been found in all three categories, the semi-specific interventions have proved to be the most efficient for improving finger flexor and upper limb strength, endurance, and RFD, as well as climbing performance across several climbing performance levels (Mundry et al., 2021; Devise et al., 2022; Vigouroux and Devise, 2024; Hermans et al., 2022; Levernier and Laffaye, 2019; Lopez-Rivera and Gonzalez-Badiloo, 2012; Medernach et al., 2015a; Stien et al., 2021a). Furthermore, the reviews (Stien et al., 2023; Langer et al., 2023) also concluded that a mix of maximal strength (i.e., 1-5 repetitions/seconds) and hypertrophy training (i.e., 8–15 repetitions/3–30 s) tended to yield the greatest effects in improving climbing-specific strength and strength endurance.

Despite a growing body of scientific literature on climbing-specific training approaches, the number of interventional studies in climbing are still limited (Stien et al., 2023; Langer et al., 2023). The studies also vary methodically (i.e., design, performance level, sex, test- and training procedures), making it challenging to compare and generalize the findings to training recommendations for a variety of climbers. Importantly, both reviews (Stien et al., 2023; Langer et al., 2023) highlighted that the findings of their meta-analyses must be interpreted with caution, and call for more intervention studies including a control condition which only a few of the previous studies have done (Mundry et al., 2021; Devise et al., 2022; Vigouroux and Devise, 2024; Hermans et al., 2022; Levernier and Laffaye, 2019; Medernach et al., 2015a; Stien et al., 2021a; Hermans et al., 2017; Medernach et al., 2015b).

Of note, most studies have only included climbing-specific tests (i.e., finger strength, finger endurance) as predictors for climbing performance whereas only a handful of studies have included actual climbing performance tests (Stien et al., 2024; Stien et al., 2021a; Hermans et al., 2017; Philippe et al., 2019; Stien et al., 2021b). In terms of training interventions, previous studies have used isometric finger contractions (typically hanging from fingerboards or rungs) while climbing includes a dynamic contraction grasping, holding on, and then moving to next hold. To the best of the authors’ knowledge, no study has yet examined the effects of dynamic finger strength training which in traditional resistance training has demonstrated effective for improving both strength and power (American College of Sports Medicine, 2009; Grgic et al., 2018; Suchomel et al., 2018; Schoenfeld et al., 2021). Finally, boulderers are also underrepresented in the existing literature, and little is known about specific resistance training for boulderers (Ozimek et al., 2017). Therefore, the aim of this study was to examine the effects of a 5-week dynamic finger strength training program on bouldering performance and climbing specific strength among advanced to elite level boulderers. It was hypothesized that including the dynamic finger strength exercises into the training program would substantially improve bouldering performance, finger strength, and RFD.

## Materials and methods

### Participants

Prior to the intervention, a sample size calculation was conducted using G*Power 3.1 (Dusseldorf, Germany) based on the strength findings by Hermans et al. (2017). With an alpha level of 0.05, and statistical power of 80%, a sample size of 16 participants in each group was necessary to detect significant differences between the groups. To be included, participants had to be at least 18 years old, free of climbing-related injuries in the past 6 months, had to boulder regularly (i.e., bouldering minimum once a week) the past 6 months, and having a self-reported bouldering red-point grade of 6B+ (IRCRA 18) or higher. The self-reported best red-point bouldering performance within the last 6 months was reported using the Font grading system (1–8A/B/C) and further converted to the numeric IRCRA scale (1–32) (Draper et al., 2015). Self-reported climbing performance have previous been demonstrated as reliable and suitable for use in scientific contexts (Draper et al., 2011). Thirty-seven participants who fulfilled the inclusion criteria volunteered for the study. Due to various reasons not related to the study, six participants dropped out (DST = 3, CON = 3) during the intervention, leaving 31 boulderers who completed the intervention. Group characteristics are shown in Table 1. Prior to pre-testing, all participants were informed about the study, and signed an informed consent form. The study procedures were evaluated by the Norwegian Agency for Shared Services in Education and Research (857,841) and approved by the local research ethics committee at the Western Norway University of Applied Sciences (23/09938–3).

**TABLE 1:** Group characteristics at baseline.

	DFS (n = 17)	CON (n = 14)
Sex	2 female, 15 male	1 female, 13 male
Bouldering performance level	17 advanced	10 advanced, 4 elite
Weight (kg)	72.1 ± 6.6	77.6 ± 11.9
Age (yr)	27.7 ± 7.2	26.8 ± 3.3
Height (cm)	179.2 ± 5.2	183.5 ± 8.7
Weekly bouldering sessions (n)	3.1 ± 0.9	2.5 ± 0.9
Bouldering experience (yr)	7.8 ± 8.2	4.8 ± 2.4
Best red-point grad (IRCRA^a^)	21.0 ± 2.9	19.4 ± 1.5

^a^ International Rock Climbing Research Association. All values are presented as mean ± standard deviation. No between group-differences were observed (*p* > 0.05).

### Study design

A randomized controlled trial was used to examine the effects of a 5-week dynamic finger strength training. Pre- and- post intervention, the participants were tested in bouldering performance, isometric finger flexor peak- and average force, RFD and 1RM dynamic finger flexor strength. After the pre-testing, the participants were randomized by drawing lots to either the dynamic finger-strength training group (DFS) or the control group (CON). Both groups continued their bouldering and training routines as usual during the intervention period.

### Testing procedures

Participants were instructed to refrain from intense climbing-related activity 48 h before testing. A standardized warm-up started each training- and testing day, consisting of 15 min easy to moderate intensity traversing and bouldering on self-selected holds while instructed to avoid fatigue. Testing was conducted in a standardized order and divided into 2 days, separated by 2–5 days. Day 1 included anthropometric measurements and an interview about their bouldering experience (years and weekly sessions), and best red-point self-reported bouldering performance in the last 6 months. Afterwards, bouldering performance was examined on a Kilter Board, before a familiarization test was conducted for the isometric pull-up and 1RM dynamic finger flexor strength tests. On day 2, maximal force and RFD of the finger flexors were tested in the isometric pull-up test, followed by the 1RM dynamic finger flexor strength test. Pre- and post-testing were identical except for the familiarization tests on day 1. Day 1 of the post-test was conducted 48–96 h after last training session. All holds used in the tests were brushed regularly, and participants were provided with chalk to ensure similar grip conditions.

### Bouldering performance

Bouldering performance was tested on the standardized system wall (12 × 12 Kilter Board, Kilter, LLC, Boulder, Colorado, United State) (Stien et al., 2024). The test consisted of 5 boulder problems with increasing difficulty individually adjusted to the participants self-reported best red-point bouldering performance. Using the randomize function on the Kilter Board application, the first boulder having at least 5 hand moves and 50 registered ascends were chosen for each degree of difficulty. Two problems were graded under their respective best red-point performance, while one problem corresponded to their best red-point performance, and two problems were graded over their best red-point bouldering performance. For instance, a boulderer with a best performance of 7A would attempt the following boulder problems on the test: 6C, 6C+, 7A, 7A+ and 7B. The steepness of the Kilter Board was set to 25-degree overhang on problems up to 7B (IRCRA: 23). As there were no boulders graded over 7B using the 25-degree overhang with 50 registered ascends, the steepness had to be increased to 40-degree overhang from 7B+ and harder (IRCRA: 24). Furthermore, the boulders below 7B grade using the 40-degree overhang involved mainly jugs holds with long distance between holds resulting in greater importance of shoulder and back strength. The included boulders are listed in Table 2.

**TABLE 2:** Overview of the boulder problems included in the performance test.

Font grade	Name of the boulder problem	Total moves	Degrees of overhang	Acsends (per 02.10.2023)
6a+	Jessica’s Brofest	5	25	90
6B	Trans-Dimensional Counsil of Ricks	7	25	220
6B+	Iwa-kakeru!	5	25	247
6C	Pinch N Crimp	6	25	67
6C+	Crimp Daddy	7	25	84
7A	Jump, stick, and a…	5	25	89
7A+	Reaching for the Bottle Again	5	25	771
7B	Bastkjaers masterpiece	6	25	218
7B+	Mimi Zilla 2.0	6	40	1768
7C	Lizardon	6	40	253
7C+	9 Holes of Jade	6	40	87
8A	Wagon Lite	8	40	798
8A+	Miami Vice Grip	7	40	165

The participants were given 4 min for each of the five boulder problems, with 3 min rest in between. Prior to each problem, participants were given a 2-minute observation time. A maximum of 3 attempts were given per boulder to limit fatigue. A total score based on the number of successful moves in the best attempt for each boulder problem was summed up. A successful move (i.e., control on the next hold) was awarded one point, while a touch without control was awarded half a point. The same person scored the pre- and post results. All participants were instructed to avoid using Kilter Board during the intervention.

### 1RM dynamic finger flexor strength

**FIGURE 1:**
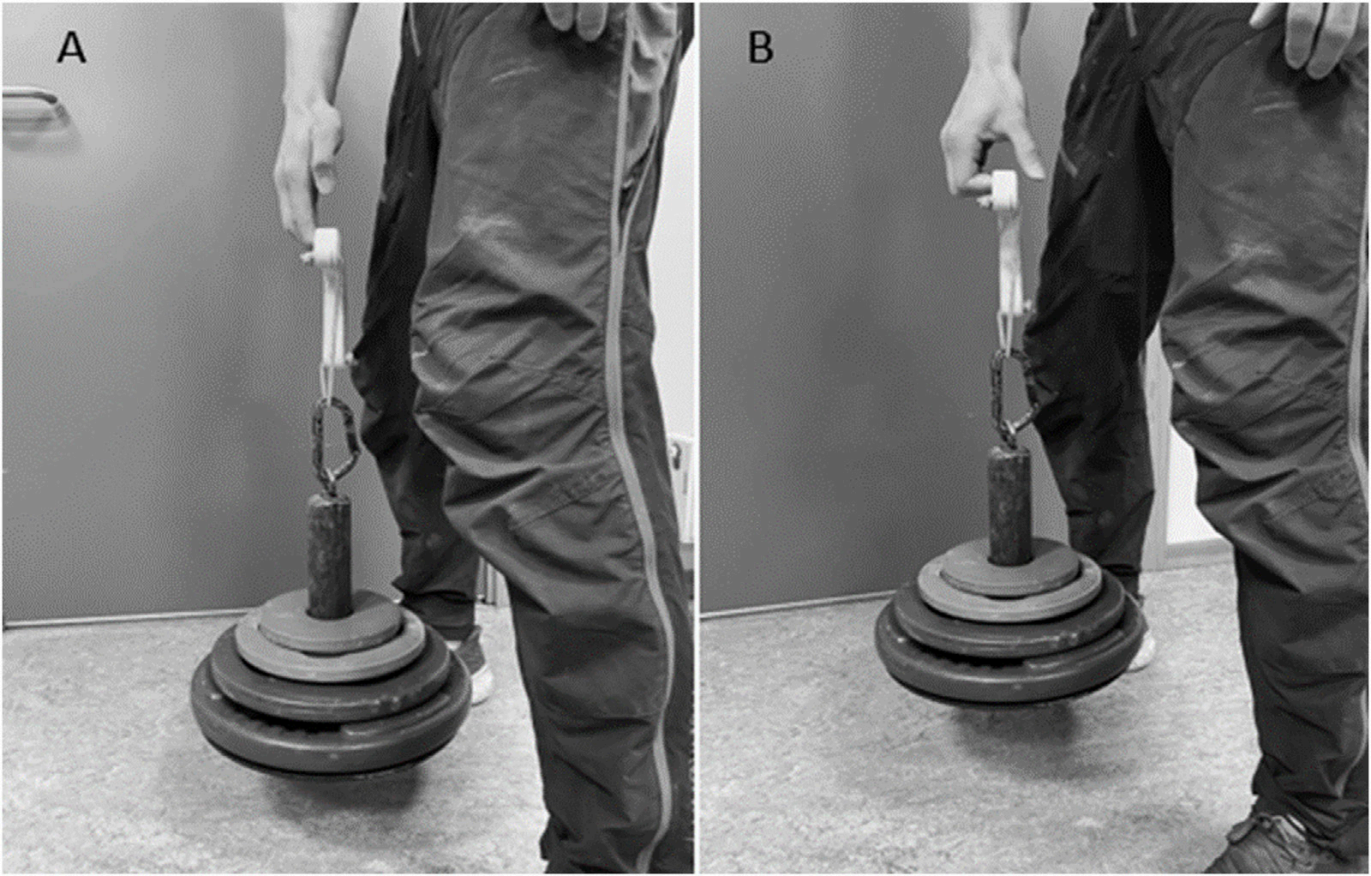
Setup for the 1RM dynamic finger flexor strength test with the starting position **(A)** and the finishing position **(B)**.

1RM dynamic finger flexor strength was tested using the dominant hand (i.e., the hand used for writing). The test was conducted on an apparatus with a 19 mm deep wooden climbing hold (Tindeq, V-rings, Trondheim, Norway) connected to a custom-made platform holding weight plates (Figure 1). Participants started the attempt upright with knees and hips extended using an open hand grip (Figure 1A) and pulled into a half crimp grip (Figure 1B) with passive thumb. For the 1RM attempt to be approved, the fingers had to be flexed to a minimum of 90° in the PIP joint without any movement in the shoulder- or elbow joint to create momentum. The test started with one repetition at the assumed 70% of 1RM (based on results from the familiarization test), followed by a weight increase of 10% for each repetition until failure. After failure, one last attempt was given at a weight in the middle of the failed attempt and the last approved attempt. Two minutes rest was given between attempts. The highest load with an approved attempt was set as 1RM. The smallest adjustment was 0.5 kg. Basen on an unpublished material from our lab, the test demonstrated an excellent intraclass correlation coefficient (ICC) of 0.989 with and acceptable coefficient of variation (CV) of 11.6% (Langer et al., 2023).

### Isometric pull-up

**FIGURE 2:**
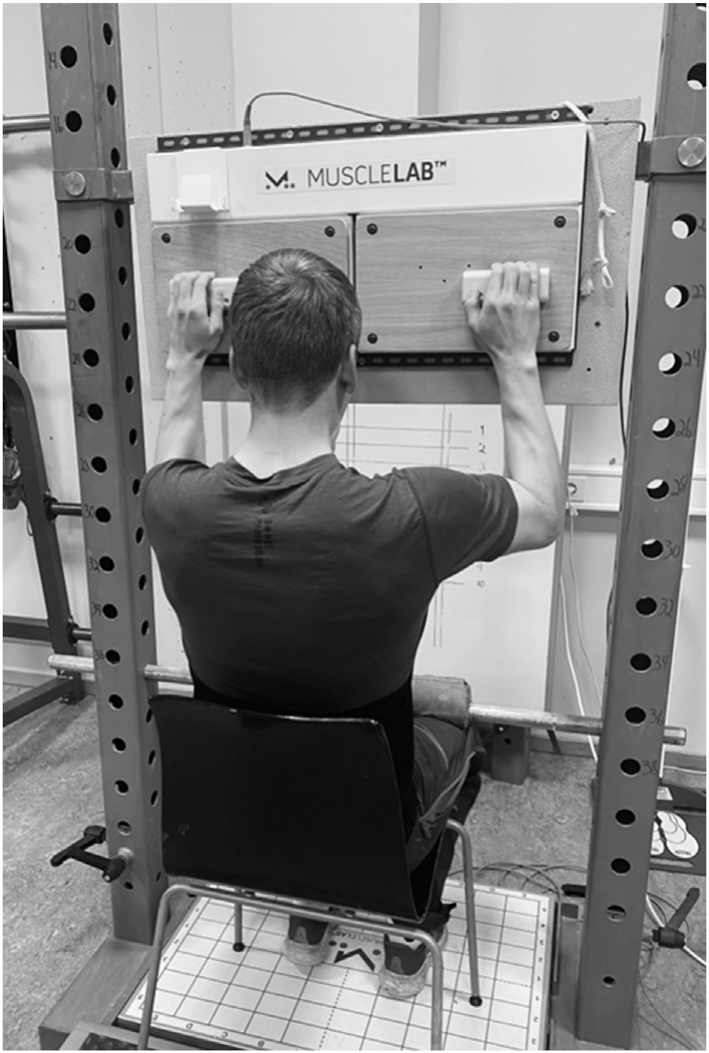
Test set-up for the isometric pull-up test.

An isometric pull-up test performed on two 23 mm deep rounded wooden rungs (Metolius Climbing, Bend, Oregon, United States), was used to measure climbing-specific maximal finger flexor strength and RFD (Vereide et al., 2022; Hermans et al., 2022; Stien et al., 2021a). The rungs were 13 cm wide and placed 36 cm apart from each other (Figure 2). The force (N) was measured through four force cells connected to the rungs with a sampling frequency of 200 Hz (Ergotest Innovation A/S, Porsgrunn, Norway). During the test, participants were seated in a chair with a barbell fixed over the upper half of their thigh, making upwards movement impossible during the attempt (Figure 2). The vertical height of the rungs was individually adjusted so that the participants had a 90° elbow joint angle (measured with a goniometer) while using both hands in a half crimp grip with passive a thumb (Figure 2). On verbal command, participants initiated the pull-up with the instructions of quickly building up to maximal force and maintain it for 5 seconds (until verbal command to stop). Maximal average force (Favg) was then set as the highest average force within a 3 s time-period (Vereide et al., 2022; Hermans et al., 2022; Stien et al., 2021b). Three minutes of rest were given between attempts. In the second condition, measuring peak force (Fpeak) and RFD, participants were instructed to pull as fast and forcefully as possible for one to 2 s (Maffiuletti et al., 2016), with 1.5 min rest between attempts. Fpeak was set as the absolute highest force output during the attempt, and RFD was set as the increase in force output during the first 200 ms from the onset of the contraction (Hermans et al., 2022; Levernier and Laffaye, 2019). The onset of the contraction was manually identified as the point when force rose with more than 5 N over 5 ms, after a steady period (Levernier and Laffaye, 2019; Stien et al., 2021b; Andersen and Aagaard, 2006). The best attempt from each variable were used in the analysis and analyzed using the commercial software (MuscleLab v.10.4, Ergotest Innovation A/S, Porsgrunn, Norway). The test results showed a CV varying from 1.98% to 6.87% based on the 3 attempts at pre-test, and an intraclass ICC ranging from 0.87–0.95 based on familiarization- and pre-test results which corresponds to good-to excellent reliability (Langer et al., 2023; Koo and Li, 2016).

### Training procedures

As all climbing involves contraction and relaxing of the finger flexors, the intervention consisted of dynamic finger strength training. The program contained 3 sessions per week and consisted of one exercise on the testing equipment used during the 1RM dynamic finger flexor strength test. The training period was 5 weeks which is correspond to a typical block in periodized training (Molmen et al., 2019; Galan-Rioja et al., 2023) and proven long enough to improve performance in climbing (Levernier and Laffaye, 2019; Stien et al., 2021a; Stien et al., 2021b). The training procedures were identical to the 1RM testing procedures described above (Figure 1). The exercise was chosen because it limits the movement to the fingers, only focusing on increasing the dynamic strength of the finger flexors.

The participants were instructed to ensure good balance, and to keep the arms and back straight throughout the set to isolate the finger flexors and minimize the risk of injury. The exercise was performed after the standardized warm-up identical as the one used before testing sessions. With the exception of the first week of training, the exercise was performed as maximal strength training (Table 3) classified by Saeterbakken et al. (2024). Each set was performed until failure with both hands, with a load individually adjusted to the number of repetitions in Table 3. The load was increased when the number of successful repetitions exceeded the target. Total duration of the dynamic finger training session was approximately 20–25 min and minimum 48 h separated each session.

**TABLE 3:** Overview of repetitions, sets and rest during the training.

Week number	Number of repetitions	Number of sets	Rest between sets (min)
1	8–10 RM	3	3
2	6–8 RM	4	3
3	4–6 RM	4	3
4	6–8 RM	5	3
5	4–6 RM	5	3

Both groups continued bouldering training and were instructed to not do any specific finger strength training (i.e., fingerboard, campus bord, etc.). Participants in both groups had to log their training in an individual Google Docs form. The form included different training methods, such as finger strength training (only for the DFS group), bouldering, endurance training, as well as duration and intensity of climbing sessions. Participants in the DFS group had to complete at least 80% of the intervention sessions to be included in the final analysis.

### Statistical analysis

All analysis were conducted using the commercial statistical software SPSS (Version 29.0, SPSS Inc., Chicago, IL, United States). Normality was assessed with the Shapiro-Wilk test, which showed that all data except 1RM dynamic finger flexor strength were normally distributed. A two-way repeated measures ANOVA was performed to assess the effect of time (pre-test and post-test) and group (intervention and control) on the dependent variables. Interaction effects between time and group were examined to determine if changes over time differed between groups (Fu and Holmer, 2015). When the ANOVA revealed significant interaction effects, paired sample t-tests were used to analyze within group changes and independent samples t-test to compare between-groups differences. Despite the non-normal distribution indicated by the Shapiro-Wilk test for the 1RM dynamic finger flexor strength data (*p* = 0.002–0.024), the skewness and kurtosis values at pre-test (skewness = 1.685, kurtosis = 4.625) and post-test (skewness = 1.033, kurtosis = 1.299) suggested that the deviations from normality were within acceptable limits for parametric testing in moderate sample sizes (Field, 2018). Additionally, variances were homogeneous between groups (pre-test variance = 45.438, post-test variance = 49.768; Levene’s Test, *p* > 0.05). Given the robustness of ANOVA to moderate violations of normality with reasonably large and equal group sizes (Glass et al., 1972; Schmider et al., 2010), and to maintain consistency in our analytical approach, we proceeded with parametric tests for this variable as well. Alpha level was set to <0.05 for statistical significance. All results are presented as mean ± standard deviation (SD) and Cohen`s d effect size (ES). The ES were calculated as the mean difference divided by the pooled and weighted standard deviations. An ES of <0.2 was considered trivial, 0.2–0.5 small, 0.5–0.8 medium and >0.8 large (Cohen, 1988).

## Results

There were no statistical differences between the groups for any of the variables at baseline (*p* = 0.43–0.86).

### Bouldering performance

**FIGURE 3:**
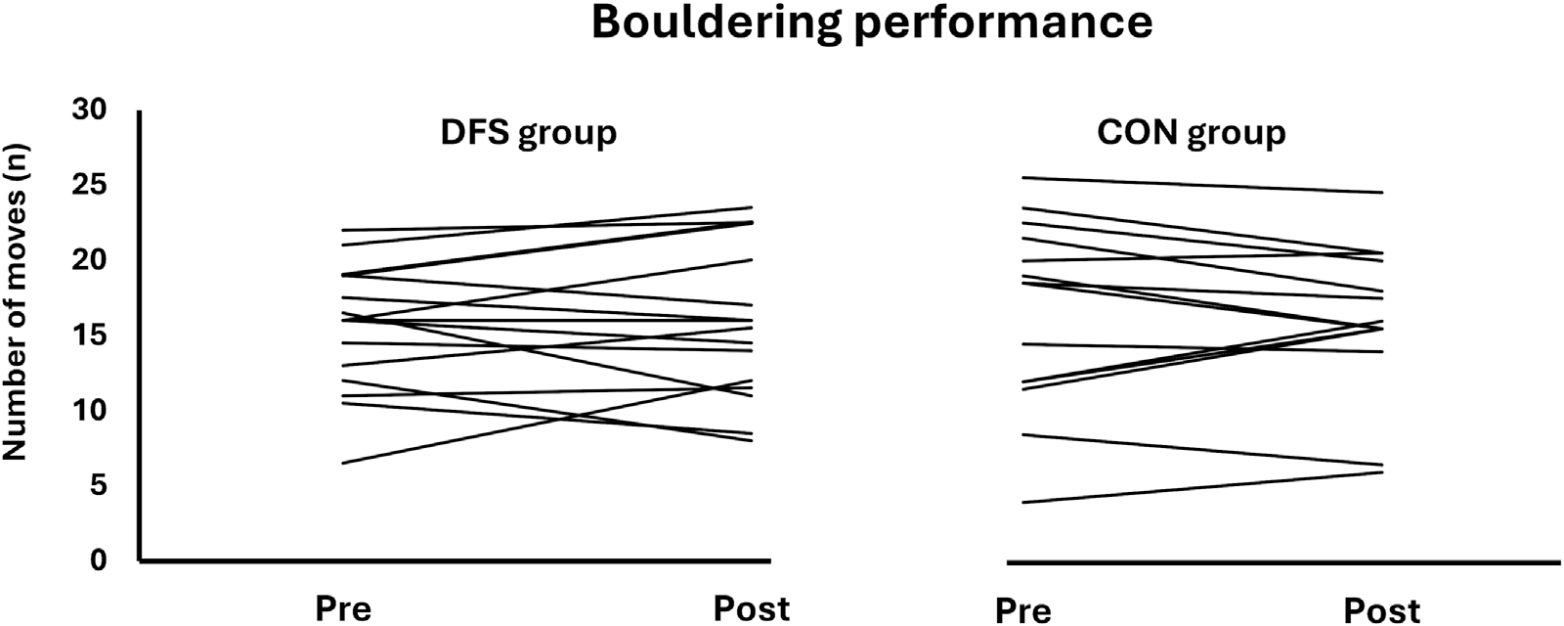
The individual pre- and post results for the bouldering performance test.

There were no statistical differences between group in bouldering performance (*p* = 0.39, ES = 0.35), nor any pre-to-post differences in the DFS group: (3.8%, *p* = 0.43, ES = 0.20) or CON group (−2.5%, *p* = 0.58, ES = −0.15) (Table 4; Figure 3).

**TABLE 4:** Pre- and post-test results in absolute values for bouldering performance, 1RM dynamic finger strength, Fpeak, Favg, RFD and weekly training sessions.

	CON (n = 14)			DFS (n = 17)		
	Pre	Post	ES	Pre	Post	ES
Bouldering performance (n)	16.5 ± 6.3	16.1 ± 5.0	-0.15	15.8 ± 4.1	16.4 ± 5.3	0.20
1RM dynamic finger strength (kg)	32.2 ± 8.3	33.9 ± 7.4^a^	0.58	33.8 ± 5.7	37.7 ± 6.7^a^	1.83
Favg (N)	848.1 ± 150.1	882.1 ± 123.7	0.56	886.4 ± 196.0	934.4 ± 197.9^a^	0.75
Fpeak (N)	917.4 ± 174.7	953.9 ± 156.8	0.41	929.0 ± 168.2	979.7 ± 186.5^a^	0.98
RFD (N/s)	3026 ± 668.5	3211 ± 643.5	0.52	3209 ± 614.0	3248 ± 603	0.14
Weekly training sessions (n)	2.5 ± 0.9	2.7 ± 0.6	0.14	3.1 ± 0.9	3.0 ± 0.7	-0.07

ES = Effect size, CON = Control group, DFS = Dynamic finger strength group. Favg = Average force, Fpeak, Peak force, RFD = rate of force development.

^a^ Significantly different from pre-test.

### 1RM dynamic finger flexor strength

**FIGURE 4:**
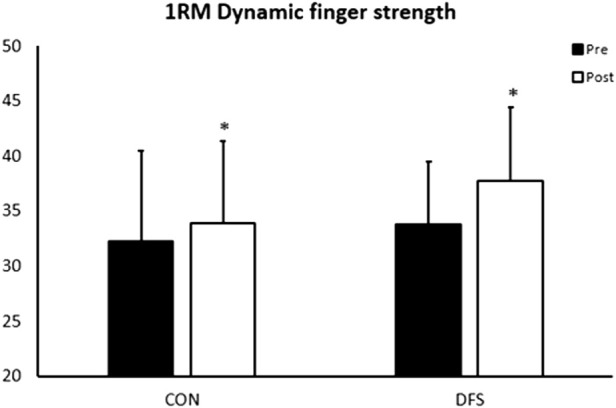
Pre- and post-test results for 1RM dynamic finger flexor strength. *Significantly different from pre-test.

**FIGURE 5:**
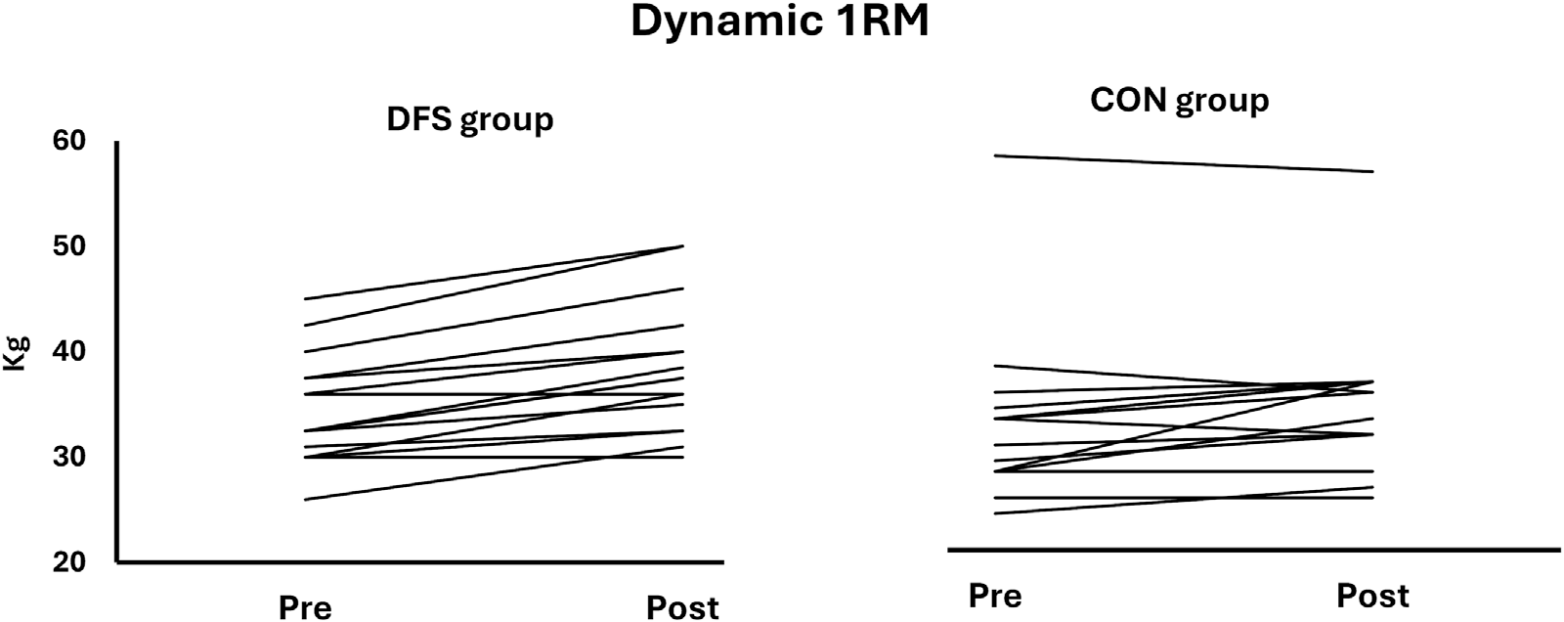
The individual pre- and post results for the dynamic 1RM test.

A significant interaction effect between time and group was found for 1RM in the dynamic finger flexor strength test (F (1, 28) = 6.024, *p* = 0.021, η^2^ = 0.117). The DFS group improved their 1RM by 11.5% (3.9 kg, *p* < 0.001, ES = 1.83), while the CON group showed a 5.3% improvement (1.7 kg, *p* = 0.048, ES = 0.58). The DFS group improved significantly more than the CON group (ES = 0.90, *p* = 0.021), but the groups were not different at pre- (ES = 0.34, *p* = 0.362) or post-test (ES = 0.67, *p* = 0.075) (Figure 4; Figure 5).

The DFS group demonstrated greater 1RM dynamic finger flexor strength than the CON group at post-test (*p* = 0.01, ES = 0.90) (Figure 4; Figure 5). Furthermore, the DFS group improved the 1RM by 11.5% equivalent to 3.9 kg (*p* < 0.01, ES = 1.83), while the CON group showed a 5.3% (1.7 kg) improvement (*p* < 0.05, ES = 0.58).

### Isometric pull-up force and RFD

The isometric pull-up test showed no statistical differences between the groups in Fpeak (*p* = 0.56, ES = 0.20), Favg (*p* = 0.42, ES = 0.22) or RFD (*p* = 0.30, ES = −0.46) (Tabel 4). The DFS group increased their force output from pre to post by 5.5% in Fpeak (*p* < 0.01, ES = 0.98) and 5.4% in Favg (*p* < 0.01, ES = 0.75) (Figure 4), whereas no statistical differences were observed in RFD (*p* = 0.57, ES = 0.14) (Table 4). The CON group showed non-significant improvements in Fpeak (*p* = 0.15, ES = 0.41), Favg (*p* = 0.06, ES = 0.56) and RFD (*p* = 0.07, ES = 0.52) from pre-to post-test (Table 4).

### Training

Number of weekly bouldering sessions during the intervention period were similar for the groups (3.1 ± 0.9 vs. 2.5 ± 0.9, *p* = 0.20, ES = 0.48), and none of the groups changed their training frequency during the intervention (DFS; 3.1 vs. 3.0 sessions per week, *p* = 0.78, ES = −0.07, and CON; 2.5 vs. 2.7 sessions per week, *p* = 0.60, ES = 0.14) (Table 4). Moreover, the training attendance in the DFS group were in average 92%.

## Discussion

The aim of the present study was to examine the effects of a 5-week dynamic finger strength (DFS) training program on bouldering performance and finger strength among boulders. There was no difference change in bouldering performance or isometric finger strength between the groups. Still, the DFS training protocol was superior compared to the CON group in percentage improving 1RM dynamic finger flexor strength, although there was only a statistical tendency (*p* = 0.075) between the groups at post-test. Both groups showed significant improvements from pre-to-post testing. Furthermore, the DFS group showed significant improvement in isometric finger strength (Fpeak and Favg) whereas the CON group remained unchanged. No within group differences were observed in RFD.

In contrast to the hypothesis, the DFS group did not improve bouldering performance. Still, the lack of bouldering improvements might be explained by numerous possible causes. In general, finger flexor strength is identified as a key factor in climbing performance (Saul et al., 2019; Stien et al., 2023; Langer et al., 2023; Saeterbakken et al., 2024). Importantly, there are multiple other factors as well (e.g., shoulder girdle strength, technique, and flexibility) determining both climbing and bouldering performance (Ginszt et al., 2023; Laffaye et al., 2016; MacLeod et al., 2007; Magiera et al., 2013; Mermier et al., 2000; Balas et al., 2012; Stien et al., 2022). Therefore, improving one determining factor in bouldering (i.e., the finger strength) may not necessarily improve performance in bouldering. Especially when the participants had over 6 years of climbing/bouldering experience and were on an advanced climbing performance level (IRCRA score of 20). Still, the present finding is supported by three of the four studies including climbing or bouldering performance as an outcome (Hermans et al., 2017; Philippe et al., 2019; Stien et al., 2021b). In contrast, Stien et al. (2021a) reported improvements in bouldering performance compared to a control condition following 5 weeks of campus board training on advanced-to elite level climbers. However, the reported pre-to post-test effect sizes were small (ES = 0.3) (Stien et al., 2021a) of which the present study displayed a comparable small effect size (ES = 0.20) for the DFS group. Of interest, the group only continuing bouldering (CON) demonstrated a negative trivial effect size (ES = −0.15) in contrast to the principle of specificity (Sale and MacDougall, 1981). It could be speculated that the randomly selection of boulders, day-to-day variation of performance may have resulted in the non-significant decrease in performance for the CON despite demonstrating a small-to moderate effects (ES = 0.41–0.58) for the dynamic and isometric finger strength. In regard of the DFS group, it could be speculated that increasing the training volume (i.e., higher training frequency and/or longer intervention period) might have proved efficient for improving bouldering performance. Short block training periods of 5 weeks have frequently been used (Stien et al., 2021a; Stien et al., 2021b; Stien et al., 2022). Still, several have argued that neural adaptations are dominate in the beginning of strength training period whereas morphological adaptations require a longer training period to be significant (Folland and Williams, 2007). The present study did not include electromyography or measurements of muscle thickness or cross section area to examine this speculation. However, the greater improvement of dynamic finger flexor strength and not in the isometric contraction in the DST group supports the task-specificity supporting the speculation of letting the neuromuscular adaptions being the dominant cause.

Similar overall training volume during the intervention period was observed between the groups. In other words, the dynamic finger training of the present study did not increase the participants overall training volume but was used as a block period to improve finger strength capacity. A 5-week period has been used in several comparable studies (Stien et al., 2024; Stien et al., 2021a; Stien et al., 2021b; Stien et al., 2022). Still, the 3 weekly sessions of dynamic finger strength training sessions (3-5 sets, 4–10 repetitions) lasting 20–25 min may not have been sufficient to increase bouldering performance. In contrast to the present study, Philippe et al. (2019) reported improved lead climbing performance following specific training methods (i.e., lead climbing and bouldering). However, Philippe et al. (2019) had a longer intervention duration (8 vs. 5 weeks), and higher training volume (5 vs. 3 weekly training sessions) compared to the present study. This speculation is supported by another study reporting that a mix of prioritized bouldering or lead climbing following a 5-week intervention with 3 weekly training sessions, did not improve performance in either discipline (Stien et al., 2021b). Finally, it’s possible that individual variations may have influenced the finding due to the relatively small sample size increasing the risk of making a type II error. Furthermore, the findings may also have been affected by the attempt restrictions in the test procedures, as some of the test boulders included relatively challenging coordination crux moves which limited the results more than finger strength. The outcomes may have been different if the included boulders focused more on finger strength (i.e., small holds without long moves). Instead, we wanted the focus on the ecological validity reflecting bouldering competition and elected boulder problems randomly from the Kilter board application.

The DFS training program was designed to increase maximal finger flexor strength based on recommendations from general- and climbing-specific resistance training (Langer et al., 2023; American College of Sports Medicine, 2009; Saeterbakken et al., 2024). In agreement with the hypothesis and these recommendations, the DFS group showed superior strength gains in the 1RM dynamic finger flexor strength test compared to the CON group. With approximately 3 folded greater effect sizes in the DFS group than the CON (ES; 1.83 vs. 0.58) this shows that the DFS training was highly effective in improving dynamic finger flexor strength. Importantly, the present finding suggest that bouldering may also be effective for improving finger flexor strength. This is partly in contrast with previous studies claiming unstructured climbing- and bouldering training to be ineffective in increasing specific strength (Balas et al., 2012; Hermans et al., 2022; Levernier and Laffaye, 2019). Still, and supported by the present findings of the CON group, several studies have observed improvements on climbing-specific tests including active control groups which continue either lead climbing or bouldering during an intervention period (Medernach et al., 2015a; Stien et al., 2021a; Hermans et al., 2017; Stien et al., 2021b). Importantly, a familiarization session was conducted to minimize potential learning effects of conducting the 1RM test and the test demonstrated excellent ICC and acceptable CV.

Typically, finger flexor strength is measured in an isometric pull-up test (Stien et al., 2022). In contrast to the hypothesis and the dynamic 1RM findings, no differences between the groups were observed. This finding is in line with most previous studies examining the effects of finger strength training among similar performance level and training duration (i.e., ≤5 weeks) (Levernier and Laffaye, 2019; Lopez-Rivera and Gonzalez-Badiloo, 2012; Medernach et al., 2015a; Stien et al., 2021b). On the other hand, two studies have reported significant finger strength improvements compared to a control group (Mundry et al., 2021; Hermans et al., 2022). Of note, these studies included climbers on a lower skill level (i.e., IRCRA <18) or conducted a training procedure of a longer duration compared to the present study, which might explain the different results. Importantly, the DFS group improved their average (ES = 0.75) and peak (ES = 0.98) isometric finger flexor strength whereas the CON group displayed statistical tendencies for increased isometric finger flexor strength with small to moderate effect sizes (ES = 0.41 and 0.56). Moreover, the different effect sizes observed for the DFS group across the finger strength tests is possibly a result of higher training specificity (Sale and MacDougall, 1981) to the 1RM test. In general, position matched dynamic and isometric strength demonstrate <50% shared variance (Lum et al., 2020). Furthermore, a recent meta-analysis displayed a two folded greater effect size in dynamic contraction (ES = 1.84) form compared to isometric contraction (ES = 0.80) after conducting a dynamic resistance training intervention (Spitz et al., 2023). It could therefore be speculated that the task-specificity of the dynamic training intervention with a limited transferability to isometric contraction form may explain the findings.

In contrast to the hypothesis, no between- or within group effects were observed in RFD. The movement velocity during the DFS training was slow due to the relatively heavy loads (i.e., 4-8RM) which may explain the findings. Interestingly, the effect size was medium in the CON group (ES = 0.52) compared to trivial effect size in the DFS group (ES = 0.14) even though no differences were observed between the groups. It could be speculated that the DFS group had a larger degree of fatigue while bouldering as the participants continued bouldering after the finger training program in contrast to the CON group. Thus, the CON group might have been able to execute more powerful boulders with higher movement velocity and quality compared to the DFS group, possibly explaining the findings. The present finding is partly in contrast to previous literature reporting improved RFD following finger strength training among both boulderers and climbers (Hermans et al., 2022; Levernier and Laffaye, 2019; Stien et al., 2021a). Moreover, Andersen and Aagaard (Andersen and Aagaard, 2006) claimed that RFD is strongly correlated with maximal voluntary contraction (MVC), indicating that the observed strength gains from the current study should have resulted in higher levels of RFD. However, Levernier and Laffaye (Levernier and Laffaye, 2019) and Stien et al. (Stien et al., 2021a) showed contradictory findings to Andersen and Aagaard (Andersen and Aagaard, 2006), as both studies reported increased RFD, while no changes was reported in maximal finger strength. Thus, the authors (Levernier and Laffaye, 2019; Stien et al., 2021a) explained the increase in RFD mainly by neural adaptions. As one-handed isometric hangs (Levernier and Laffaye, 2019) and campus board training (Stien et al., 2021a) requires high RFD to establish on shallow rungs, these methods likely offer a greater stimulus to increase RFD compared to the DFS training in the present study. As the latter stages of the RFD are mostly associated with MVC (Andersen and Aagaard, 2006), it could also be speculated that including a RFD measurement from the entire force curve would have shown different results (Vereide et al., 2022).

Despite the current study being the first to examine the effects of dynamic finger strength training on boulderers, it has its limitations. With a relatively small sample size included in the study, the statistical power is low. A larger sample size would have decreased the risk of doing a type-II error and thus strengthened the present findings. The sample only included advanced to elite boulderers, but the relatively large standard deviations from the test results show a heterogeneous sample. Moreover, both sexes were included in the sample, but the distribution was far from even with only three females. Therefore, the findings should be interpreted with caution, and may not be generalizable to females or boulderers of other performance levels. Also, the present testing procedures also has some notable limitations. Bouldering performance is challenging to measure in a reliable way. Even though testing on the Kilter board increases the reproducibility and thus allows for comparisons in other facilities, some of the included test boulders may not have been suitable for testing. For instance, some of the boulders were of uneven difficulty with an obvious crux-sequence, and two boulders shared the same starting moves (i.e., 7C and 8A + boulders). Of note, only one participant was tested on these boulders. However, the included boulders were graded by at least 50 ascends, ensuring the grade to be correct. The choice of including boulders graded under, on and over the performance level of each individual also controlled for any misreporting of the participants performance level. In addition, this choice made sure no participant was able to top all the boulders at pre-test, leaving room for progression. Of note, we did not monitor or controlled the climbing sessions conducted during the intervention period. Instead, the climbers used an individual self-reported form included climbing style, frequence, intensity, and duration. Furthermore, we only quantified boulders performance according to competition rules and we cannot exclude possible benefits of technique (Stien et al., 2024). Finally, we conducted the post-test 48–96 h after the last training session without a follow-up period. We can only speculate, but it`s possible that a post-test 2–4 weeks after the intervention may have altered the results.

The aim of the present study was to examine the effects of dynamic finger strength training on bouldering performance as well as climbing-specific strength and RFD among boulderers. Thus, more research is needed to confirm these findings and examine the effects among other performance levels. Still, the present study is one of few including an actual bouldering performance test, whereas most studies have only included climbing-specific tests (e.g., finger strength test). Despite improvements in finger flexor strength, no effects were observed in bouldering performance in the present study.

## Conclusion

The 5-week dynamic finger strength training program was not superior to bouldering in improving bouldering performance, RFD or isometric finger flexor strength. These findings suggest that dynamic finger strength training is a highly effective supplement to bouldering training for improving maximal finger strength among advanced to elite level boulderers. However, improving strength alone does not seem sufficient for improving bouldering performance. The present findings contribute to the evidence-based knowledge about short-term effects of finger strength training in bouldering and could be used by trainers and athletes for improving specific finger strength.

## Data Availability

The original contributions presented in the study are included in the article/supplementary material, further inquiries can be directed to the corresponding author.

## References

[B1] American College of Sports Medicine (2009). American College of Sports Medicine position stand. Progression models in resistance training for healthy adults. Med. Sci. Sports Exerc 41 (3), 687–708. 10.1249/MSS.0b013e3181915670 19204579

[B2] Andersen L. L. Aagaard P. (2006). Influence of maximal muscle strength and intrinsic muscle contractile properties on contractile rate of force development. Eur. J. Appl. Physiol. 96 (1), 46–52. 10.1007/s00421-005-0070-z 16249918

[B3] Balas J. Pecha O. Martin A. J. Cochrane D. (2012). Hand-arm strength and endurance as predictors of climbing performance. Eur. J. Sport Sci. 12 (1), 16–25. 10.1080/17461391.2010.546431

[B4] Cohen J. (1988). Statistical power analysis for the behavioral sciences. 2nd ed. Hillsdale, NJ; Hove: Lawrence Erlbaum.

[B5] Devise M. Lechaptois C. Berton E. Vigouroux L. (2022). Effects of different hangboard training intensities on finger grip strength, stamina, and endurance. Front. Sports Act. Living 4, 862782. 10.3389/fspor.2022.862782 35498522 PMC9039162

[B6] Draper N. Dickson T. Blackwell G. Fryer S. Priestley S. Winter D. (2011). Self-reported ability assessment in rock climbing. J. Sports Sci. 29 (8), 851–858. 10.1080/02640414.2011.565362 21491325

[B7] Draper N. Giles D. Schöffl V. Konstantin Fuss F. Watts P. Wolf P. (2015). Comparative grading scales, statistical analyses, climber descriptors and ability grouping: international Rock Climbing Research Association position statement. Sports Technol. 8 (3-4), 88–94. 10.1080/19346182.2015.1107081

[B8] Draper N. Giles D. Taylor N. Vigouroux L. España-Romero V. Baláš J. (2021). Performance assessment for rock climbers: the international rock climbing research association sport-specific test battery. Int. J. Sports Physiol. Perform. 16 (9), 1242–1252. 10.1123/ijspp.2020-0672 33652414

[B9] Fanchini M. Violette F. Impellizzeri F. M. Maffiuletti N. A. (2013). Differences in climbing-specific strength between boulder and lead rock climbers. J. Strength Cond. Res. 27 (2), 310–314. 10.1519/JSC.0b013e3182577026 22505133

[B10] Field A. (2018). Discovering statistics using IBM SPSS statistics. 5th ed. Los Angles, London, New Delhi, Singapore, Wasingthon DC, Melbourne: Sage Publications. Available at: http://repo.darmajaya.ac.id/5678/1/Discovering%20Statistics%20Using%20IBM%20SPSS%20Statistics%20%28%20PDFDrive%20%29.pdf

[B11] Folland J. P. Williams A. G. (2007). The adaptations to strength training: morphological and neurological contributions to increased strength. Sports Med. 37 (2), 145–168. 10.2165/00007256-200737020-00004 17241104

[B12] Fryer S. Stone K. J. Sveen J. Dickson T. España-Romero V. Giles D. (2017). Differences in forearm strength, endurance, and hemodynamic kinetics between male boulderers and lead rock climbers. Eur. J. Sport Sci. 17 (9), 1177–1183. 10.1080/17461391.2017.1353135 28753391

[B13] Fu R. Holmer H. K. (2015). Change score or followup score? An empirical evaluation of the impact of choice of mean difference estimates. Rockville (MD): Agency for Healthcare Research and Quality. 25927135

[B14] Galan-Rioja M. A. Gonzalez-Ravé J. M. González-Mohíno F. Seiler S. (2023). Training periodization, intensity distribution, and volume in trained cyclists: a systematic review. Int. J. Sports Physiol. Perform. 18 (2), 112–122. 10.1123/ijspp.2022-0302 36640771

[B15] Ginszt M. Saito M. Zięba E. Majcher P. Kikuchi N. (2023). Body composition, anthropometric parameters, and strength-endurance characteristics of sport climbers: a systematic review. J. Strength Cond. Res. 37 (6), 1339–1348. 10.1519/JSC.0000000000004464 36930882 PMC10212580

[B16] Glass G. V. Peckham P. D. Sanders J. R. (1972). Consequences of failure to meet assumptions underlying the fixed effects analyses of variance and covariance. Rev. Edu Res. 42 (3), 237–288. 10.3102/00346543042003237

[B17] Grgic J. Schoenfeld B. J. Davies T. B. Lazinica B. Krieger J. W. Pedisic Z. (2018). Effect of resistance training frequency on gains in muscular strength: a systematic review and meta-analysis. Sports Med. 48 (5), 1207–1220. 10.1007/s40279-018-0872-x 29470825

[B18] Hermans E. Andersen V. Saeterbakken A. H. (2017). The effects of high resistance-few repetitions and low resistance-high repetitions resistance training on climbing performance. Eur. J. Sport Sci. 17 (4), 378–385. 10.1080/17461391.2016.1248499 27863457

[B19] Hermans E. Saeterbakken A. H. Vereide V. Nord I. S. O. Stien N. Andersen V. (2022). The effects of 10 Weeks hangboard training on climbing specific maximal strength, explosive strength, and finger endurance. Front. Sports Act. Living 4, 888158. 10.3389/fspor.2022.888158 35571743 PMC9092147

[B20] Koo T. K. Li M. Y. (2016). A guideline of selecting and reporting intraclass correlation coefficients for reliability research. J. Chiropr. Med. 15 (2), 155–163. 10.1016/j.jcm.2016.02.012 27330520 PMC4913118

[B21] Laffaye G. Collin J. M. Levernier G. Padulo J. (2014). Upper-limb power test in rock-climbing. Int. J. Sports Med. 35 (8), 670–675. 10.1055/s-0033-1358473 24554556

[B22] Laffaye G. Levernier G. Collin J. M. (2016). Determinant factors in climbing ability: influence of strength, anthropometry, and neuromuscular fatigue. Scand. J. Med. Sci. Sports 26 (10), 1151–1159. 10.1111/sms.12558 26453999

[B23] Langer K. Simon C. Wiemeyer J. (2023). Strength training in climbing: a systematic review. J. Strength Cond. Res. 37 (3), 751–767. 10.1519/JSC.0000000000004286 36820707

[B24] Levernier G. Laffaye G. (2019). Four weeks of finger grip training increases the rate of force development and the maximal force in elite and top world-ranking climbers. J. Strength Cond. Res. 33 (9), 2471–2480. 10.1519/JSC.0000000000002230 28945641

[B25] Lopez-Rivera E. Gonzalez-Badiloo J. (2012). The effects of two maximum grip strength training methods using the same effort duration and different edge depth on grip endurance in elite climbers. Sports Technol. 5 (3-4), 100–110. 10.1080/19346182.2012.716061

[B26] Lum D. Haff G. G. Barbosa T. M. (2020). The relationship between isometric force-time characteristics and dynamic performance: a systematic review. Sports (Basel) 8 (5), 63. 10.3390/sports8050063 32429176 PMC7281606

[B27] MacLeod D. Sutherland D. L. Buntin L. Whitaker A. Aitchison T. Watt I. (2007). Physiological determinants of climbing-specific finger endurance and sport rock climbing performance. J. Sports Sci. 25 (12), 1433–1443. 10.1080/02640410600944550 17786696

[B28] Maffiuletti N. A. Aagaard P. Blazevich A. J. Folland J. Tillin N. Duchateau J. (2016). Rate of force development: physiological and methodological considerations. Eur. J. Appl. Physiol. 116 (6), 1091–1116. 10.1007/s00421-016-3346-6 26941023 PMC4875063

[B29] Magiera A. Roczniok R. Maszczyk A. Czuba M. Kantyka J. Kurek P. (2013). The structure of performance of a sport rock climber. J. Hum. Kinet. 36, 107–117. 10.2478/hukin-2013-0011 23717360 PMC3661882

[B30] Medernach J. P. Kleinoder H. Lotzerich H. H. (2015a). Fingerboard in competitive bouldering: training effects on grip strength and endurance. J. Strength Cond. Res. 29 (8), 2286–2295. 10.1519/JSC.0000000000000873 26203738

[B31] Medernach J. P. Kleinoder H. Lotzerich H. H. (2015b). Effect of interval bouldering on hanging and climbing time to exhaustion. Sports Technol. 8 (3-4), 76–82. 10.1080/19346182.2015.1063643

[B32] Mermier C. M. Janot J. M. Parker D. L. Swan J. G. (2000). Physiological and anthropometric determinants of sport climbing performance. Br. J. Sports Med. 34 (5), 359–365. 10.1136/bjsm.34.5.359 11049146 PMC1756253

[B33] Molmen K. S. Ofsteng S. J. Ronnestad B. R. (2019). Block periodization of endurance training - a systematic review and meta-analysis. Open Access J. Sports Med. 10, 145–160. 10.2147/OAJSM.S180408 31802956 PMC6802561

[B34] Mundry S. Steinmetz G. Atkinson E. J. Schilling A. F. Schöffl V. R. Saul D. (2021). Hangboard training in advanced climbers: a randomized controlled trial. Sci. Rep. 11 (1), 13530. 10.1038/s41598-021-92898-2 34188125 PMC8241953

[B35] Ozimek M. Krawczyk M. Zadarko E. Barabasz Z. Ambroży T. Stanula A. (2017). Somatic profile of the elite boulderers in Poland. J. Strength Cond. Res. 31 (4), 963–970. 10.1519/JSC.0000000000001673 28328714

[B36] Philippe M. Filzwieser I. Leichtfried V. Blank C. Haslinger S. Fleckenstein J. (2019). The effects of 8 weeks of two different training methods on on-sight lead climbing performance. J. Sports Med. Phys. Fit. 59 (4), 561–568. 10.23736/S0022-4707.18.08399-8 29722250

[B37] Saeterbakken A. H. Stien N. Pedersen H. Langer K. Scott S. Michailov M. L. (2024). The connection between resistance training, climbing performance, and injury prevention. Sports Med. Open 10 (1), 10. 10.1186/s40798-024-00677-w 38240903 PMC10798940

[B38] Sale D. MacDougall D. (1981). Specificity in strength training: a review for the coach and athlete. Can. J. Appl. Sport Sci. 6 (2), 87–92. 7016357

[B39] Saul D. Steinmetz G. Lehmann W. Schilling A. F. (2019). Determinants for success in climbing: a systematic review. J. Exerc Sci. Fit. 17 (3), 91–100. 10.1016/j.jesf.2019.04.002 31193395 PMC6527913

[B40] Schmider E. Ziegler M. Danay E. Beyer L. Bühner M. (2010). Is it really robust? Reinvestigating the robustness of ANOVA against violations of the normal distribution assumption. Metodology 6 (4), 147–151. 10.1027/1614-2241/a000016

[B41] Schoenfeld B. J. Grgic J. Van Every D. W. Plotkin D. L. (2021). Loading recommendations for muscle strength, hypertrophy, and local endurance: a Re-examination of the repetition continuum. Sports (Basel) 9 (2), 32. 10.3390/sports9020032 33671664 PMC7927075

[B42] Spitz R. W. Kataoka R. Dankel S. J. Bell Z. W. Song J. S. Wong V. (2023). Quantifying the generality of strength adaptation: a meta-analysis. Sports Med. 53 (3), 637–648. 10.1007/s40279-022-01790-0 36396899

[B43] Stien N. Frøysaker T. F. Hermans E. Vereide V. A. Andersen V. Saeterbakken A. H. (2021b). The effects of prioritizing lead or boulder climbing among intermediate climbers. Front. Sports Act. Living 3, 661167. 10.3389/fspor.2021.661167 33969299 PMC8100213

[B44] Stien N. Langer K. Andersen V. Engelsrud G. H. Olsen E. Saeterbakken A. H. (2024). Development of specific motor skills through system wall bouldering training: a pilot study. Transl. Sports Med. 2024, 5584962. 10.1155/2024/5584962 39015173 PMC11250695

[B45] Stien N. Pedersen H. Vereide V. A. Saeterbakken A. H. Hermans E. Kalland J. (2021a). Effects of two vs. Four weekly campus board training sessions on bouldering performance and climbing-specific tests in advanced and elite climbers. J. Sports Sci. Med. 20 (3), 438–447. 10.52082/jssm.2021.438 34267583 PMC8256519

[B46] Stien N. Riiser A. Shaw M. P. Saeterbakken A. H. Andersen V. (2023). Effects of climbing- and resistance-training on climbing-specific performance: a systematic review and meta-analysis. Biol. Sport 40 (1), 179–191. 10.5114/biolsport.2023.113295 36636194 PMC9806751

[B47] Stien N. Saeterbakken A. H. Andersen V. (2022). Tests and procedures for measuring endurance, strength, and power in climbing-A mini-review. Front. Sports Act. Living 4, 847447. 10.3389/fspor.2022.847447 35308594 PMC8931302

[B48] Stien N. Saeterbakken A. H. Hermans E. Vereide V. A. Olsen E. Andersen V. (2019). Comparison of climbing-specific strength and endurance between lead and boulder climbers. PLoS One 14 (9), e0222529. 10.1371/journal.pone.0222529 31536569 PMC6752829

[B49] Suchomel T. J. Nimphius S. Bellon C. R. Stone M. H. (2018). The importance of muscular strength: training considerations. Sports Med. 48 (4), 765–785. 10.1007/s40279-018-0862-z 29372481

[B50] Vereide V. Andersen V. Hermans E. Kalland J. Saeterbakken A. H. Stien N. (2022). Differences in upper-body peak force and rate of force development in male intermediate, advanced, and elite sport climbers. Front. Sports Act. Living 4, 888061. 10.3389/fspor.2022.888061 35837246 PMC9274001

[B51] Vigouroux L. Devise M. (2024). Pull-up performance is affected differently by the muscle contraction regimens practiced during training among climbers. Bioeng. (Basel) 11 (1), 85. 10.3390/bioengineering11010085 PMC10813506 38247962

